# STIM Proteins and Regulation of SOCE in ER-PM Junctions

**DOI:** 10.3390/biom12081152

**Published:** 2022-08-20

**Authors:** Moaz Ahmad, Sasirekha Narayanasamy, Hwei Ling Ong, Indu Ambudkar

**Affiliations:** Secretory Physiology Section, National Institute of Dental and Craniofacial Research, National Institutes of Health, Bethesda, MD 20892, USA

**Keywords:** ER-PM junctions, STIM, Orai1, phospholipids, calcium signaling, SOCE

## Abstract

ER-PM junctions are membrane contact sites formed by the endoplasmic reticulum (ER) and plasma membrane (PM) in close apposition together. The formation and stability of these junctions are dependent on constitutive and dynamic enrichment of proteins, which either contribute to junctional stability or modulate the lipid levels of both ER and plasma membranes. The ER-PM junctions have come under much scrutiny recently as they serve as hubs for assembling the Ca^2+^ signaling complexes. This review summarizes: (1) key findings that underlie the abilities of STIM proteins to accumulate in ER-PM junctions; (2) the modulation of Orai/STIM complexes by other components found within the same junction; and (3) how Orai1 channel activation is coordinated and coupled with downstream signaling pathways.

## 1. Introduction

Membrane contact sites formed by juxtaposed endoplasmic reticulum (ER) membrane and plasma membrane (PM) are known as ER-PM junctions. These junctions have been under much scrutiny recently [1,2,3,4,5,6,7], given their importance as hubs for assembling ion channels together with their regulatory and modulatory proteins to form different signaling complexes. Phosphatidylinositol lipids (e.g., phosphatidylinositol 4,5-bisphosphate (PIP_2_)) are involved in various cellular processes [8,9], such as ion transport, membrane trafficking, and cytoskeleton dynamics. Other components that are constitutively or dynamically enriched at ER-PM junctions include extended synaptotagmins (E-Syts) [7,10], stromal interaction molecules (STIMs) [5,11,12], TMEM24 [13], Kv2 voltage-gated K^+^ channels, oxysterol-binding protein (OSBP)-related proteins (ORP) 5 and ORP8, and GRAM domain-containing proteins (GRAMDs) [14]. Some of these are structural and scaffolding proteins that contribute to junctional stability, while others are lipid modulators involved in the replenishment and turnover of membrane lipids within the junctions. Among the proteins associated with ER-PM junctions, the STIM proteins are of critical importance in Ca^2+^ signaling as they are the sole regulators of Orai channels that mediate store-operated Ca^2+^ entry (SOCE) [15,16,17]. ER-PM junctions are the site of interaction between STIMs and Orai channels as well as the coupling of Orai1-mediated Ca^2+^ entry with regulation of downstream targets and cellular functions, e.g., Ca^2+^-dependent activation of nuclear factor of activated T-cells 1 (NFAT1)-mediated gene expression [18,19]. An increasing number of findings in recent years have demonstrated that these ER-PM junctions serve as centers for assembly of Ca^2+^ signaling complexes. 

Ca^2+^ signaling in cells is triggered in response to agonist stimulation of cell surface receptors coupled to activation of PLC and cause hydrolysis of PIP_2_, IP_3_ generation and IP_3_-triggered Ca^2+^ release from the ER-Ca^2+^ store via activation of IP_3_R. STIM1 and STIM2 are single transmembrane domain ER proteins that sense changes in ER Ca^2+^ store via their N-terminal Ca^2+^ binding EF-hand domains [20,21]. While STIM1 responds to substantial depletion of ER-Ca^2+^ due to its higher Ca^2+^ binding affinity, STIM2 senses smaller changes in [Ca^2+^]_ER_ due to its relatively lower affinity for Ca^2+^ [11,22]. Both STIMs work in concert to regulate the activation of SOCE in response to varying stimulus intensities [5,23]. Extensive studies with STIM1 have revealed that during basal cellular conditions, it tracks along the microtubules by interacting with EB1/EB3 proteins at the plus ends [24]. Sufficient decrease in [Ca^2+^]_ER_ results in dissociation of Ca^2+^ from the luminal EF hand-SAM domains, which triggers a conformational change in the protein causing it to oligomerize and cluster in ER-PM junctions [25]. In this activated conformation, STIM1 binds to PIP_2_ at the PM via its C-terminal polybasic domain (PBD). This interaction of STIM1 with lipids stabilizes the protein clusters. Once clustered, the STIM-Orai1 activation region (SOAR) can bind to and recruit Orai1 into the ER-PM junctions, where it gates the channel to induce SOCE. Thus, the site of SOCE is within ER-PM junctions. In contrast, STIM2 displays pre-clustering in the ER-PM junctions in unstimulated cells [22]. In addition to the relatively lower affinity of its EF hand for Ca^2+^, the C-terminal PBD domain of STIM2 also has a higher affinity for PM PIP_2_ as compared to that of STIM1 (further details provided below). Other studies demonstrated that unlike STIM1, STIM2 is in a partially activated confirmation in resting cells and can thus bind to and activate Orai1 to trigger Ca^2+^ influx in absence of overt agonist stimulation [5,11,22,23]. While STIM1 is a strong activator of Orai1, STIM2 induces relatively weaker activation of the channel. However, due to its lower Ca^2+^ affinity, it responds efficiently to low [agonist]. Under these conditions, it also recruits STIM1 to the ER-PM junctions and promotes STIM1 clustering, STIM1/Orai1 assembly and strong channel function even when ER-Ca^2+^ store depletion is not sufficient to be sensed by STIM1. Thus, STIM2 is an efficient coordinator of Ca^2+^ signaling as it can tune the agonist sensitivity of SOCE [5,22,23].

This review highlights the key properties of STIM proteins that underlie their abilities to accumulate in ER-PM junctions and describes how other components, such as actin cytoskeleton, PM PIP_2_, and other accessory proteins, modulate the function of Orai/STIM complexes. We will also discuss assembly of a signaling complex that not only co-ordinates Orai1 activation, but also the coupling of Orai1-mediated Ca^2+^ signals to the activation of downstream signaling pathways. 

## 2. Properties and Function of the STIM Polybasic Domains

The PBD of STIM1 and STIM2 play a crucial role in their function by localizing the proteins within ER-PM junctions which facilitates their interaction with, and gating of, Orai1 channel in the PM [26,27,28,29]. A STIM1 mutant lacking the PBD (aa 671-685, STIM1ΔK) fails to form puncta following ER-Ca^2+^ store depletion, unless co-expressed with Orai1. In a later study, deleting the terminal 5 amino acids (aa 829-833) of STIM2-PBD (STIM2ΔK5) was found to be sufficient to adversely impact puncta formation [22] 

Primary structure analysis reveals that the C-termini of STIM1 and STIM2 contain eight (7 Lys (K) and 1 Arg (R)) and nine Lys (K) basic amino acids residues, respectively. The basic amino acids are well separated by hydrophobic residues. It is hypothesized that the adjacent hydrophobic residues in this domain might stabilize the PIP_2_-PBD interaction by inserting their side chains into the membrane hydrophobic core as observed in other proteins [30,31]. Although the PBDs of STIMs 1 and 2 are highly similar in sequence they have different biophysical and biochemical properties. Secondary structure analysis as determined by circular dichroism spectra revealed that the PBD of STIM2 forms a well-ordered α-helical structure in the presence of trifluoroethanol, which mimics hydrophobic membrane environments [26,32]. In addition, it has been shown that in STIM2, this domain forms an amphipathic α-helix with the basic residues on one side and the bulky side chains of the hydrophobic residues facing the other side. Disrupting the hydrophobic patches using site-directed mutagenesis results in reduced binding to PIP_2_- and phosphatidylinositol (3,4,5)-trisphosphate (PIP_3_)-containing liposomes [27]. Conversely, STIM1-PBD exists in a random coil-like structure and remains unstructured in the presence of hydrophobic medium [26]. Differences in the secondary structure of PBDs in both STIM proteins have been associated with two conserved proline (Pro) (helix breakers) residues, which are present in STIM1 but absent in STIM2. Together, these studies indicate that the absence of Pro residues in STIM2 promotes the formation of short amphipathic helices that aids in its binding of phosphoinositides at the PM, a similar characteristic feature observed in lipid binding signal of yeast Ist2 [26,27,33,34,35]. 

The PBD of both STIMs contain positively charged K residues that interact electrostatically with acidic phospholipids in the PM, such as PIP_2_ and PIP_3_. Polybasic sequences are embedded between the hydrophilic and hydrophobic regions of the membrane, which allows basic and hydrophobic side chains to interact with acidic lipid phosphate groups and acyl chains, respectively [30,36,37]. Disrupting the interactions between STIM-PBD and PM PIP_2_ disrupted the assembly and function of Orai1/STIM1 complexes. Depletion of PM PIP_2_ using a rapamycin-inducible 5′-phosphatase decreases the clustering of both STIMs and Orai1, as well as SOCE (no effect on Ca^2+^ release from ER-Ca^2+^ stores) [19,38]. Liposome binding assays indicate that the purified C-terminal region of STIM2 binds PIP_2_-containing liposomes with a very high affinity as compared to STIM1 (K*_D_* for STIM2: 3.3 ± 0.8 μM; K*_D_* for STIM1: 8 ± 2 μM). It is interesting to note that while STIM1 binds only PIP_2_, STIM2 binds both PIP_2_ and PIP_3_ [27]. The differences in their affinity and selectivity to phosphoinositides could be due to their different oligomerization properties. It has been shown that the K-rich domain of STIM1 is unable to oligomerize without the CC (coiled coil) domain. Conversely, the K-rich domain of STIM2 forms an oligomer independent of its CC domain. This is due to the unique presence of Cysteine (Cys) residues in STIM2 (but not in STIM1) located upstream of the K-rich domain. Mutating the Cys residues to Ala or reducing Cys in the K-rich domain have been shown to abolish PIP_2_ binding [26]. The relatively lower affinity of STIM2 for Ca^2+^ (K*_D_*: 0.5 mM) at the N-terminus, together with the high PIP_2_ binding activity at the C-terminus, could contribute to its regulation under basal conditions that causes pre-clustering and activation of Orai1 in absence of exogenous stimulation [11,26,39]. On the other hand, a high affinity for Ca^2+^ (K_D_: 0.2 mM) and low binding of STIM1 K-rich domain to PM lipids underlie the poor activation of STIM1 by minor fluctuations in the [Ca^2+^]_ER_ levels [26,39]. 

Both the STIM1 and STIM2 contain many predicted/putative Ca^2+^/calmodulin (CaM)- binding sites in their cytoplasmic domains including the K-rich domain [40]. Isothermal titration calorimetry studies reveal that K-rich domain of the STIM proteins bind CaM with very high affinity in the presence of Ca^2+^ (K*_D_*: 0.8 μM for STIM1 and 0.9 μM for STIM2). Whereas, in the absence of Ca^2+^, STIM1 and STIM2 binds CaM with lower affinity (K*_D_* = 55 μM and 150 μM, respectively) [41]. These studies suggest that the cytosolic [Ca^2+^] levels modulate the STIM-CaM interaction and the Ca^2+^-induced exposure of hydrophobic surfaces of CaM might interact with the hydrophobic residues present in the PBD of STIM proteins, as observed in many CaM target proteins [41,42,43]. Since the binding of CaM interferes with the interaction of STIM2-PBD to liposomes in a Ca^2+^- and PIP_2_-dependent manner, it is most likely that CaM competes with phosphoinositides to bind the K-rich domain at high cytosolic [Ca^2+^] levels and thereby, destabilize the ER-PM contacts and downregulate STIM2-mediated Ca^2+^ influx [26,41]. Future studies should examine this possible mechanism in greater detail to identify the exact binding site of CaM as well as the physiological consequences of this interaction. 

## 3. Modulation of SOCE by Plasma Membrane PIP_2_, Actin, and Septins

Activation of SOCE and downstream Ca^2+^-dependent effectors have been shown to be affected by reorganization of the actin cytoskeleton, although there have been conflicting reports regarding this. A possible basis for the involvement of actin in SOCE could be plasma membrane PIP_2_ interaction with, and modulation of, various actin-binding proteins (ABPs). An increase in plasma membrane PIP_2_ levels induces actin filament assembly, while a reduction promotes disassembly [44,45]. These ABPs have been shown to associate with the plasma membrane via electrostatic interactions without penetrating the lipid bilayer or binding specifically to any regions of the plasma membrane [46]. Different ABPs exhibit varying affinities and binding dynamics with plasma membrane PIP_2_. While cofilin displays transient and low-affinity interactions with phosphoinositide-rich membranes, N-WASP maintain its interaction at lower phosphoinositide densities for longer periods of time. Ezrin, which links the actin cytoskeleton to the plasma membrane, displays high-affinity interactions at lower phosphoinositide densities [46]. The diversity of interactions between ABPs and plasma membrane PIP_2_ enables the cells to calibrate the activities of signaling proteins and ion channels in response to physiological stimulation. 

As recently reviewed in [45], stimulation of immune cells leads to reorganization of the cortical actin cytoskeleton in order to transduce signals from the plasma membrane receptor to intracellular sites. Treatments with compounds that either promote actin depolymerization (e.g., lantrunculins A and B (Lat A and B respectively) and cytochalasin D (CytD) or stabilize the actin cytoskeletal network (e.g., jasplakinolide) have induced contrasting effects on SOCE in immune cells. Lat A inhibited anti-CD3-induced Ca^2+^ responses and significantly decrease phospholipase C (PLC) activity in TALL-104 human leukemic cytotoxic T-lymphocytes [47] and Jurkat T cells [48], whereas CytD enhanced Ca^2+^ responses and IP_3_ levels in IgE-sensitized RBL-2H3 cells [49] and murine B lymphocytes [50,51]. In turn, elevated [Ca^2+^]_i_ can also regulate assembly and disassembly of the actin filaments. Following the formation of an immune synapse between T cells and antigen-presenting cells, STIM1/Orai1 complexes start forming at the periphery and then migrate to the center of the synapse [52]. Orai1-mediated Ca^2+^ influx then promotes actin depolymerization by localizing Wiskott-Aldrich syndrome protein family verprolin-homologous protein 2 (WAVE2) to the periphery of the lamellipod, where WAVE2 initiates actin nucleation via ARP2/3. This results in a highly visible ring-like formation of cortical actin towards the edge of the immune synapse. Retrograde actin flow continues to push the STIM1/Orai1 complexes to the synapse center, where puncta density was highest but actin filament density was the lowest. This increases the density of Ca^2+^ signals at the center of the immune synapse, which creates a positive feedback loop to maintain SOCE [52]. 

**Figure 1:**
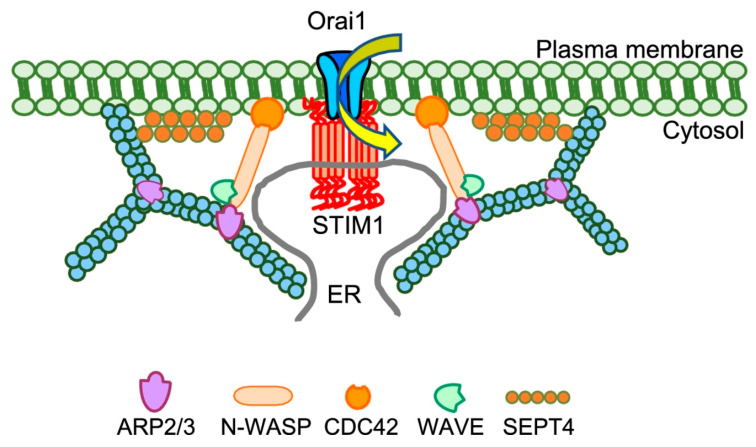
**Cytoskeletal remodeling in ER-PM junctions.** Remodeling of the actin cytoskeleton and septin filaments to enable formation and stabilization of ER-PM junctions populated by STIM1/Orai1 signaling complex [38].

Septin 4 (SEPT4), a member of the Septin 2 subgroup, modulates the clustering of Orai1 and STIM1 at the ER-PM junctions by rearranging PIP_2_ at the junctional plasma membrane [53,54,55]. Knocking down SEPT4 inhibited both thapsigargin (Tg)-induced SOCE and nuclear translocation of NFAT in Jurkat and HeLa cells [53]. SEPT4 relocalizes to the plasma membrane and has been shown to regulate the formation of ER-PM junctions and enhance the stability of the STIM1/Orai1 complexes in HeLa cells [55]. This raises the possibility that SEPT4 requires accessory proteins or lipids to mediate its effects on STIM1/Orai1. Septins functionally interact with and regulate PIP_2_-dependent proteins involved in cytoskeletal remodeling, such as cell division cycle 42 (CDC42). CDC42 mediates local actin remodeling by activating actin-related proteins 2/3 and the Wiskott-Aldrich syndrome protein (WASP) family of proteins [56,57]. Following store depletion in HEK293 cells, actin remodels to form a ring-like structure around emerging STIM1 puncta, which likely supports the assembly of Orai1/STIM1 complex within the ER-PM junctions [38]. Knockdown of SEPT4, CDC42, WASP, WASP-family verprolin-homologous protein (WAVE) and ARP2/3, attenuated SOCE and reduced nuclear translocation of NFAT1 by adversely affecting STIM1 and Orai1 clustering. Integrity of the actin cytoskeletal network was also disrupted by these knockdowns. Depletion of the ER-Ca^2+^ stores induced recruitment of CDC42 to the subplasma membrane region, which was decreased by loss of SEPT4 and depletion of plasma membrane PIP_2_. Therefore, remodeling of the actin cytoskeleton within the ER-PM junctions is coordinated by SEPT4 and plasma membrane PIP_2_ via the functions of CDC42, N-WASP, WAVE and ARP2/3 proteins (Figure 1) [38]. Together, the assembly of proteins that mediate the fast remodeling of actin can dynamically contribute to the assembly of STIM1/SOCE complex and likely affect the architecture of the ER-PM junctions itself. Detailed structural analysis will be needed to determine changes in the junctions and how that might impact SOCE and cell function. 

Septin 7 (SEPT7), the sole member of the Septin 7 subgroup interacts with plasma membrane PIP_2_ via its N-terminal polybasic region in human neural progenitor cells [58]. Unlike SEPT4, loss of SEPT7 had no effect on SOCE following stimulation with thapsigargin (Tg), but significantly increases constitutive Ca^2+^ entry via Orai1 in unstimulated cells. Nonetheless, STIM1 is still required for constitutive Orai1 channel activity. The authors proposed that knocking down SEPT7 may have disrupted the assembly of septins near the plasma membrane, which forms a barrier to prevent STIM1 from interacting with and gating Orai1 in the absence of cell stimulation [58]. Similar results were also reported for SEPT4 in an earlier study. Additionally, treatment with forchlorfenuron, which induced hyperpolymerization of septin filaments, impaired recruitment of Orai1 into and co-localization with STIM1 in the ER-PM junctions and significantly dampened SOCE [53]. A diffusion trap process has been proposed to explain the formation of STIM1/Orai1 complexes in the ER-PM junctions. Briefly, activated STIM1 diffuses from the ER and gets trapped in the junctions through its interactions with the plasma membrane [59] via the PBD domains [26,27]. The trapped STIM1 subsequently recruits Orai1 and also gates the channel to trigger Orai1-mediated extracellular Ca^2+^ entry [59,60]. 

Remodeling of the actin cytoskeleton has also been shown to reciprocally regulate plasma membrane PIP_2_. Treatment with Lat B, an actin polymerization inhibitor, impaired the translocation of Nir2 to the ER-PM junctions, suppressing the replenishment of plasma membrane PIP_2_ following receptor-induced hydrolysis by phospholipase C (PLC). Enrichment of cortical actin drives the formation of lipid rafts in the plasma membrane, within which PIP_2_ is compartmentalized and enriched [61,62,63]. Extended synaptotagmins (E-Syts), mammalian homologues of yeast tricalbins, tether the ER membrane to the plasma membrane in a PIP_2_-dependent manner [10,33]. The mammalian E-Syt family consists of three members, E-Syts 1–3, which are localized in the ER membrane. All E-Syts have C2 domains that interact with the plasma membrane PIP_2_–C2C for E-Syts 2 and 3, C2C and C2E for E-Syt1. While E-Syts 2 and 3 are constitutive ER-PM tethers, E-Syt1 requires Ca^2+^ binding to its C2A and C2C domains to facilitate its interaction with PIP_2_. Binding of Ca^2+^ to C2A releases the autoinhibitory interaction between the C2A and synaptotagmin domains to facilitate the lipid transport mediated by E-Syt1, while binding to C2C enables both C2C and C2E domains to interact with plasma membrane PIP_2_ [64]. E-Syt1-mediated ER-PM tethering facilitates the recruitment of Nir2 to the plasma membrane where it replenishes PIP_2_ following G-protein coupled histamine receptor-induced hydrolysis [7]. Knocking down all E-Syts significantly reduced the number of ER-PM junctions but interestingly, did not affect clustering of STIM1 and Orai1, as well as SOCE. Ultrastructural analysis using immunogold labeling revealed that thin cortical ER near the PM were less affected by the triple E-Syt knockdown, when compared to the predominantly wide ER tubules. As such, the authors proposed that the presence of thin ER is sufficient for the clustering of STIM1 and Orai1 to mediate SOCE [10]. However, it was unclear whether E-Syt1 populates the same ER-PM junctions as STIM1 and Orai1. Fluorescent microscopy studies showed that E-Syt1 only partially co-localizes with STIM1 after ER-Ca^2+^ store depletion in HeLa cells [65], while cryo-electron tomography revealed distinct membrane contact sites (MCSs) formed by E-Syt1 and STIM1 in COS-7 cells [66]. A recent study using super-resolution imaging [67] reported that extracellular Ca^2+^ influx via SOCE activates E-Syt1 translocation to the ER-PM junctional region. Although E-Syt1 does not constitute the MCSs per se, it is involved in reorganizing neighboring ER structures into ring-shaped MCSs which enclose the E-Syt1 puncta. This helps to stabilize MCSs and accelerate local ER Ca^2+^ replenishment. Overall, these findings demonstrate different roles of STIM1 and E-Syt1 in MCS formation regulation, SOCE activation and ER Ca^2+^ store replenishment. 

In addition to the involvement of actin cytoskeleton, there is also a link between microtubules and SOCE. STIM1 directly binds to the microtubule plus end tracking protein EB1 and forms EB1-dependent comet-like accumulations at the sites where polymerizing microtubule ends come in contact with the ER network. Depolymerization of microtubules with nocodazole caused a change from a fibrillar STIM1 localization to one that was similar to that of the ER, which was accompanied by decrease in SOCE. This led to the suggestion that microtubule-STIM1 association might have a role in regulating STIM1 activity and SOCE [68]. Further studies demonstrated that STIM1-microtubule interaction might be involved in ER-remodeling. STIM1 overexpression strongly stimulates ER extension occurring through the microtubule “tip attachment complex” (TAC) mechanism, a process whereby an ER tubule attaches to and elongates together with the EB1-positive end of a growing microtubule. Depletion of STIM1 and EB1 decreases TAC-dependent ER protrusion, indicating that microtubule growth-dependent concentration of STIM1 in the ER membrane plays a role in ER remodeling [69]. Nevertheless, the role of the STIM1–EB1 interaction in regulating SOCE remains unresolved. It has been suggested that EB1 binding constitutes a trapping mechanism restricting STIM1 targeting to ER–PM junctions. This limits STIM1 translocation to ER–PM junctions during ER Ca^2+^ depletion and prevents excess SOCE and ER Ca^2+^ overload. These authors suggest that STIM1–EB1 interaction shapes the kinetics and amplitude of local SOCE in cellular regions with growing MTs and contributes to spatiotemporal regulation of Ca^2+^ signaling crucial for cellular functions and homeostasis [70]. Another interesting finding pertaining to regulation of STIM1-EB1 interaction is phosphorylation of STIM1 via activation of ERK1/2 following ER-Ca^2+^ store depletion. ERK1/2-dependent phosphorylation of STIM1 Ser575, Ser608, and Ser621 triggers the dissociation of STIM1 from EB1 and facilitates STIM1 clustering leading to SOCE activation. Conversely, the replenishment of Ca^2+^ stores induces STIM1 dephosphorylation via as yet unknown phosphatase(s) which causes reassociation of STIM1 with EB1 and the microtubule localization pattern of STIM1 [71]. 

## 4. Accessory Proteins Modulating STIMs and SOCE

Novel binding partners will likely to be discovered as additional techniques are used to detect protein-protein interactions that occur either transiently or over a longer period of time. Previous studies have used various techniques, such as immunoaffinity purification (e.g., TMEM20/POST [72], CRAC2A [73], junctate [74], thrombospondin-1 [75], surfeit locus protein 4 [76] and ER membrane protein complex 1 (EMC1) [77]), functional siRNA screens (e.g., SARAF [78] and TMEM110 [79]), and glutathione-S-transferase pull-down assay (e.g., EB-1 and EB-3 proteins [69], junctophilin-4 [80] and Golli [81]). Proteomic mapping utilizing an engineered ascorbate peroxidase (APEX2) fused to STIM1 identified STIMATE as a positive regulator of STIM1 [82]. While the APEX2 approach enables detection of transient protein-protein interactions occurring within a short period of time, the proximity-labeling technique detects interactions occurring over longer time periods and was recently used to identify gelsolin (GSN) as a binding partner for STIM1. GSN functions as an actin-severing protein, in a Ca^2+^-dependent manner, to promote assembly and disassembly of actin filaments. Due to the close proximity of GSN near STIM1, the local Ca^2+^ microdomain generated near the mouth of Orai1 channels following STIM1 gating and SOCE activation has been proposed to be the source of high [Ca^2+^]_i_ required for the actin-severing activity of GSN [83]. Note that a majority of the previously identified partners for STIM1 interact with the protein via its cytoplasmic C-terminus [84]. A technique utilizing purified recombinant N-terminus of STIM1 (EF hand and sterile-α-motif domains) immobilized to BIACore sensor chips identified the ER oxidoreductase, ERp75, as a negative SOCE regulator [85]. A recent study using immunoaffinity purification of STIM1 identified EMC1 as a positive regulator for SOCE, which interacts with STIM1 via its N-terminal regions in the ER lumen [77].

A recent study used ContactID, a new in situ system that labels nearby (<20 nm) proteins in an ATP-dependent manner using pBirA, a mutated biotin protein ligase from *Escherichia coli* [86]. To ensure the detection of only proteins within the ER-PM junctions, each half of pBirA was fused to STIM1 and Orai1, respectively. Following ER-Ca^2+^ store depletion, aggregation of STIM1 and Orai1 at these junctions reconstituted pBirA, which then converted biotin to biotin-AMP to label nearby proteins. Using this method, the authors identified progesterone receptor membrane component 1 (PGRMC1) as a positive regulator of the STIM1/Orai1 complex and SOCE. While PGRMC1 resided in the ER in unstimulated cells, the protein colocalized with STIM1 and Orai1 in the ER-PM junctions following Tg-induced ER-Ca^2+^ store depletion. Loss of PGRMC1 by shRNA treatment or genetic depletion drastically reduced both SOCE and NFAT-dependent luciferase activity. PGRMC1 facilitates the translocation of STIM1 from the ER towards the plasma membrane as loss of the protein significantly delayed STIM1 translocation and puncta formation, and reduced STIM1-dependent recruitment of Orai1 following ER-Ca^2+^ store depletion. PGRMC1 directly binds to the CC1-CAD region of STIM1 to facilitate the conformational switching required to activate STIM1. 

## 5. STIM2 Response to ER-Ca^2+^ Depletion Is a Checkpoint for SOCE Activation

As discussed above, the EF hand domain of STIM2 has a low Ca^2+^ affinity that enables the protein to respond to minimal decreases in [Ca^2+^]_ER_ [11,23,82,87,88]. When overexpressed in cells, STIM2 displays constitutive clustering within ER-PM junctions where it recruits and activates Orai1 channels, causing Ca^2+^ entry in unstimulated cells [11,88,89]. Furthermore, pre-clustering of STIM2 promotes recruitment of Orai1/STIM1 into the junctions and facilitates STIM1 activation under conditions when [Ca^2+^]_ER_ is not sufficiently depleted [23,82]. These previous data suggest an important role for pre-clustered STIM2 in regulating Orai1 function under various stimulus intensities. There is, however, little information regarding the molecular mechanisms, or cellular cues, that regulate clustering of endogenous STIM2 in cells. A particular concern is that exogenous overexpression of STIM2 could alter the stoichiometry of endogenous STIM/Orai complexes, which might artificially force them into the junctions to cause pre-clustering. A recent study examined endogenous STIM2 and its regulation under physiological conditions by using a gene editing technique to knock-in mVenus tag and generate cells expressing fluorescently tagged endogenous STIM2 [5]. This study showed that endogenous STIM2 is pre-clustered in the ER-PM junctional region of cells under basal conditions. Importantly, while majority of STIM2 clusters are mobile, there is a small population of relatively immobile STIM2 clusters. Further, immobilization of native STIM2 clusters is triggered by decreases in local [Ca^2+^]_ER_ that are mediated by functional (also referred to as “licensed”) IP_3_ receptors (IP_3_R) and sensed by STIM2 N-terminus. These licensed receptors represent a small population of IP_3_Rs in a cell and have been shown to be relatively immobile [90]. These IP_3_Rs localized within the ER-PM junctional region are specialized and proposed to serve as first responders to agonist-induced PIP_2_ hydrolysis. Their localization allows them to sense initial, local, increases in IP_3_ and thus initiate ER-Ca^2+^ depletion. The findings presented by Ahmad et al. [5] suggest that STIM2 pre-clustering is strongly linked to this pool of IP_3_R. They show that constitutive PIP_2_-PLC activity, together with cAMP/protein kinase A (PKA) signaling, determines IP_3_R function under basal resting conditions of the cell. Consistent with this response of STIM2 at ambient stimuli, there is an increase in immobile STIM2 clusters following simulation of cells with a Ca^2+^-mobilizing agonist. Further, the immobile STIM2 clusters demarcate sites where Orai1 and STIM1 cluster with STIM2 following agonist stimulation [5].

**Figure 2:**
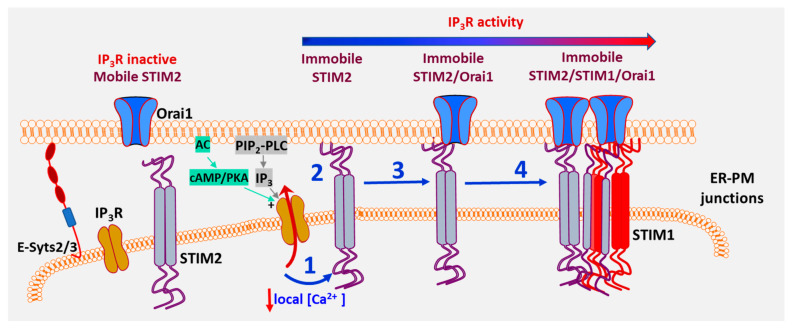
Functional communication between IP_3_R and STIM2 at subthreshold stimuli is a critical checkpoint for initiation of SOCE. The model refers to cellular responses under ambient and low-intensity stimuli. IP_3_R and STIM2 are localized in the ER–PM junctional region. Under ambient conditions (without agonist addition), constitutive PLC-dependent PIP_2_ hydrolysis as well as cAMP/PKA activity regulate IP_3_R activity. When STIM2 is in the vicinity of a functional IP_3_R, it senses the lower [Ca^2+^]_ER_ (1), which leads to scaffolding to the plasma membrane and immobilization (2). Orai1 is then recruited to immobile STIM2 (3) and with further [Ca^2+^]_ER_ decrease STIM1 is also recruited to immobile STIM2 (4) [5].

While STIM2 pre-clusters are dependent on E-Syts2/3, which serve as constitutive tethers linking ER with the PM [10], these proteins are not sufficient for STIM2 pre-clustering. A major finding of this study [5] was that IP_3_R channel activity within sub-plasma membrane ER of cells controls the constitutive clustering of STIM2. The close proximity of STIM2 and IP_3_R clusters allows the luminal N-terminus of STIM2 to sense IP_3_R-mediated decrease in local [Ca^2+^]_ER_, leading to immobilization of STIM2 in ER-PM junctions. Notably, both Orai1 and STIM1 are stabilized within ER-PM junctions when co-clustered with the relatively immobile STIM2 and the number of stable complexes is increased in a dose- and time-dependent manner after agonist stimulation (see model in Figure 2). Thus, immobilization of STIM2 clusters in response to local [Ca^2+^]_ER_ decrease marks a critical checkpoint for SOCE initiation. This change in STIM2 clustering is also the first step in coupling ER-Ca^2+^ store depletion with SOCE activation [5]. It is interesting to note that STIM2 forms complexes with ryanodine receptor, STIM1 and Orai1 in T cells, which determine generation of Ca^2+^ microdomains following T cell receptor stimulation [91]. Conversely, Ca^2+^ binding N-terminal region of STIM proteins is suggested to suppress IP_3_R function under resting conditions. This inhibition is relieved when STIMs are activated with increasing agonist concentrations [92]. A mathematical model described to explain the spontaneous Ca^2+^ microdomains that are detected in unstimulated T cells [93] proposes that pre-formed clusters of STIM2/Orai1 are localized in close proximity to IP_3_Rs, which facilitates STIM2 to detect reduction in local [Ca^2+^]_ER_ caused by spontaneous IP_3_R activity. While further studies are required to determine the exact factors that govern localization of functional IP_3_R near ER-PM junctions, these recent findings reveal that STIM2 serves as the crucial first responder that senses the decrease in local [Ca^2+^]_ER_ mediated by IP_3_Rs. Thus, pre-clustering of STIM2 in ER-PM junctions of unstimulated cells is not a random event but rather, it is orchestrated by IP_3_R function via decrease in local [Ca^2+^]_ER_ which is sensed by the STIM2 N-terminus. When a mobile STIM2 cluster is present in the vicinity of an activated “licensed” IP_3_R [90], it senses the decrease in local [Ca^2+^]_ER_ and responds causing scaffolding of its C-terminal polybasic domain to PM PIP_2_. This results in immobilization of the STIM2 cluster. Both Orai1 and STIM1 converge on these immobile STIM2 clusters and once co-clustered with STIM2, these proteins also display decreased mobility [5]. This critical functional link between IP_3_R and STIM2 underlies the constitutive clustering of STIM2 and ensures the coupling of ER-Ca^2+^ store release events with activation of SOCE and regulation of Ca^2+^ signaling in the cell. 

## 6. Assembly of Ca^2+^-Signaling Proteins in ER-PM Junctions and Regulation of Cell Function

As discussed above, increase in cytosolic Ca^2+^ resulting from Orai1 activation regulates various cellular functions including immune response, ion channel function, secretion, and Ca^2+^-dependent gene expression via activation of NFAT1. Among these, the coupling of Orai1-mediated Ca^2+^ entry with activation of NFAT1, which triggers its nuclear translocation, has been widely studied [94]. Following STIM-activation of Orai1 in response to ER-Ca^2+^ store depletion, the local Ca^2+^ increase near the mouth of the channel is sensed by CaM, which activates calcineurin to cause dephosphorylation of NFAT1 (phosphorylated NFAT1 is the inactive state). This triggers a translocation of NFAT to the nucleus where it regulates the Ca^2+^-dependent gene expression pathway. Recent studies suggest that ER-PM junctions where SOCE proteins, Orai1/STIMs are assembled play a critical role in coupling Ca^2+^ entry with NFAT1 activation. The junctions serve as sites for recruitment of various protein that relay Orai1-mediated Ca^2+^ signals to downstream targets [19,94]. The signaling components involved in NFAT1 activation are all localized near the Orai1 channel such that Ca^2+^ signals generated by the channel can be locally sensed and transduced. The relevance of a spatial organization of calcium signaling was demonstrated in a study which showed that a constitutively active Orai1 mutant failed to trigger NFAT1 activation. However, when the mutant channel was recruited to the ER-PM junction by STIM1, Ca^2+^-dependent NFAT1 activation was restored [95]. This suggests that the molecular components involved in coupling Orai1-mediated Ca^2+^ entry with NFAT1 activation are localized in a specific microdomain and that Orai1 needs to be located within that cellular domain for the downstream processes to be regulated. Indeed, further studies showed that Orai1 has to assemble with AKAP79 within ER-PM junctions, a multifunctional signaling scaffold involved in cAMP as well as NFAT1 signaling pathways [19,94,96,97]. Calcineurin, which is a key component of the NFAT1 activation cascade, is scaffolded to AKAP79. The interaction between Orai1 and AKAP79 enables localization of the channel in close proximity to calcineurin, such that Ca^2+^ entering via the channel can be sensed locally by CaM. Activated CaM in turn activates calcineurin. Importantly, AKAP79 also directly tethers NFAT1, which localizes the transcription factor within the same microdomain as the Orai1 channel complex and near calcineurin [18]. Together, this allows rapid and specific coupling between Orai1 channel function and activation of NFAT1. 

A recent study also identified an important role for STIM2 in promoting the coupling of Orai1 with NFAT1 activation [19]. As noted above, STIM2 has been shown to promote STIM1/Orai1 clustering at low stimulus intensities where ER-Ca^2+^ store depletion is not enough to trigger a robust STIM1 response [22]. In contrast, when the ER-Ca^2+^ store is substantially depleted (e.g., following stimulation by cyclopiazonic acid) knockdown of STIM2 reduces SOCE and STIM1/Orai1 clustering by about 20%. Surprisingly, under these conditions, NFAT1 activation is drastically decreased (by about 60%) [19]. This unique function of STIM2 was also shown in earlier studies with STIM2-deficient T cells, where STIM2 minimally affected Orai1/STIM1 assembly and activation of SOCE but had a distinct role in NFAT1 activation [98]. The underlying mechanism was revealed in a study reported by Son et al. [19], which demonstrated that STIM2-dependent recruitment of Orai1/STIM1 to ER-PM junctions promotes the assembly of Orai1 with AKAP79. Knockdown of STIM2 reduced NFAT1 activation despite minor effects on Orai1/STIM1 clustering as well as global or local increase in [Ca^2+^]_i_. This results from a disruption of Orai1/AKAP79 interaction in absence of STIM2. Importantly, Ca^2+^ entry mediated via assembly of Orai1/STIM1ΔK was similar both globally and locally to that mediated by Orai1/STIM1, yet it was poorly coupled with NFAT1 activation [19]. Under these conditions, Orai1 did not assemble with AKAP79 following ER-Ca^2+^ store depletion. Co-expression of STIM2 with this complex restored assembly of Orai1-AKAP79 together with enhancement of NFAT1 activation [19]. These findings suggest that STIM1 alone is not sufficient for recruitment of Orai1 to the AKAP79 signaling complex. Further, the PIP_2_-binding polybasic C-terminus of STIM2 appears to have a role in this targeting as switching the polybasic domain in STIM1 with that of STIM2 allowed efficient NFAT1 activation even when STIM2 was knocked down [19]. Although the identity and location of domains/ER-PM junctions where STIM2 clusters and recruits Orai1 and STIM1 are yet to be determined, it can now be suggested that association with STIM2 targets Orai1/STIM1 complex to a functionally relevant domain. 

AKAP79 also interacts with adenylyl cyclases and PKA. In neuronal cells, this complex appears to be localized near plasma membrane Ca^2+^ channels. Since Orai1 assembles with AKAP79, it is reasonable to assume that PKA-dependent signaling pathways can also be activated downstream of Orai1 function or involved in Orai1-mediated downstream signaling. Indeed, Orai1-mediated adenylyl cyclase activation within ER-PM junctions has been previously reported [99]. Conversely, cAMP signaling could modulate assembly and activation of Orai1 channel [97]. AKAP79 was found to be preferentially linked with Orai1 and necessary for PKA-mediated fast Ca^2+^-dependent inactivation of the channel [97]. Although more research is needed to determine whether STIM2 is essential for SOCE-dependent AC8 activation, it can be speculated that Orai1 targeting by STIM2 to the AKAP79 complex may also contribute to Ca^2+^ -dependent fast inactivation of the channel. STIM2 may thus have bifunctional effects on Orai1 channel activity regulation, enhancing function at low stimulus intensities by promoting Orai1/STIM1 clustering and STIM1 activation, while controlling cAMP-dependent fast inactivation of Orai1 when the channel is maximally activated at high stimulus intensities [22,23]. Additionally, the cAMP signaling pathway could contribute to SOCE via modulating agonist-sensitivity of Ca^2+^ signaling. In vivo, cells receive multiple signaling inputs involving several cell surface receptors. It has been previously reported that cAMP and IP_3_ exert synergistic effects on IP_3_R function [100,101]. Phosphorylation of specific amino acid residues in IP_3_R by PKA enhances the function of the channel induced by low levels of IP_3_ [100]. Thus, increasing cAMP generation (e.g., via β2 adrenergic receptor stimulation) in the presence of low IP_3_-generating [agonist], results in IP_3_R function that is greater than the additive effect of the two stimuli and substantially greater than the effect of either stimulus alone. This is due to a modulatory effect of the PKA-dependent phosphorylation on channel gating by IP_3_. Functional IP_3_Rs are relatively immobile and localized in the ER-PM junctional region, close to the site where STIM2 and STIM1 clusters are scaffolded to the PM [90]. Further, these IP_3_R are poised to detect local signals such as IP_3_ that are generated in response to the stimulation of plasma membrane receptors. 

Since STIM2 and STIM1 activation under physiological conditions is triggered in response to agonist-stimulation of IP_3_ generation and ER-Ca^2+^ release via IP_3_R, enhancement of ER-Ca^2+^ release at low stimulus intensities can lead to increased STIM clustering, Orai1 recruitment and SOCE even at low [agonist]. As noted above, the spatial arrangement between STIM2 and IP_3_R, or STIM1 and IP_3_R, determines the efficiency of sensing decrease in ER-Ca^2+^. Based on our recent findings [5], we can predict that at low stimulus intensities, such as those involved in the synergistic effects of cAMP+IP_3_ on IP_3_R, STIM2 might have a major role in conveying the ER-Ca^2+^ status to ER-PM junctions for enhancement of Orai1 function and SOCE. This coordinated regulation of STIM proteins and Orai1 within ER-PM junctions results in efficient tuning of Orai1 channel activity and regulation of cell function across varying stimulus intensities and multiple signaling inputs. 

## 7. Conclusions

In aggregate, the STIM/Orai1 signaling complex interacts with a multitude of proteins and lipids to stabilize the ER-PM junctions within which these complexes and their interacting partners are localized. The actin cytoskeleton, septins, and plasma membrane lipids function as lateral diffusion barriers for compartmentalizing and stabilizing STIM/Orai1 complexes into discrete Ca^2+^ signaling microdomains. The efficient recruitment of Orai1 to STIM in these junctions ensures spatio-temporal specificity in generating agonist-induced and store-dependent Ca^2+^ responses. This in turn allows for the efficacious transduction of Ca^2+^ signals to downstream events, such as the nuclear translocation of NFAT and activation of gene expression.

## Data Availability

Not applicable.
